# The Effect of Tailored, Daily, Smartphone Feedback to Lifestyle Self-Monitoring on Weight Loss at 12 Months: the SMARTER Randomized Clinical Trial

**DOI:** 10.2196/38243

**Published:** 2022-07-05

**Authors:** Lora E Burke, Susan M Sereika, Zhadyra Bizhanova, Bambang Parmanto, Jacob Kariuki, Jessica Cheng, Britney Beatrice, Maribel Cedillo, I Wayan Pulantara, Yuhan Wang, India Loar, Molly B Conroy

**Affiliations:** 1 School of Nursing University of Pittsburgh Pittsburgh, PA United States; 2 School of Public Health University of Pittsburgh Pittsburgh, PA United States; 3 School of Rehabilitation Sciences University of Pittsburgh Pittsburgh, PA United States; 4 School of Medicine University of Utah Salt Lake City, UT United States

**Keywords:** self-monitoring, behavioral intervention/weight loss, feedback messages, engagement, mHealth, adherence, obesity, randomized clinical trial, smart scales, physical activity trackers, digital health

## Abstract

**Background:**

Self-monitoring (SM) is the centerpiece of behavioral weight loss treatment, but the efficacy of smartphone-delivered SM feedback (FB) has not been tested in large, long-term, randomized trials.

**Objective:**

The aim of this study was to establish the efficacy of providing remote FB to diet, physical activity (PA), and weight SM on improving weight loss outcomes when comparing the SM plus FB (SM+FB) condition to the SM-only condition in a 12-month randomized controlled trial. The study was a single-site, population-based trial that took place in southwestern Pennsylvania, USA, conducted between 2018 and 2021. Participants were smartphone users age ≥18 years, able to engage in moderate PA, with a mean BMI between 27 and 43 kg/m^2^.

**Methods:**

All participants received a 90-minute, one-to-one, in-person behavioral weight loss counseling session addressing behavioral strategies, establishing participants’ dietary and PA goals, and instructing on use of the PA tracker (Fitbit Charge 2), smart scale, and diet SM app. Only SM+FB participants had access to an investigator-developed smartphone app that read SM data, in which an algorithm selected tailored messages sent to the smartphone up to 3 times daily. The SM-only participants did not receive any tailored FB based on SM data. The primary outcome was percent weight change from baseline to 12 months. Secondary outcomes included engagement with digital tools (eg, monthly percentage of FB messages opened and monthly percentage of days adherent to the calorie goal).

**Results:**

Participants (N=502) were on average 45.0 (SD 14.4) years old with a mean BMI of 33.7 (SD 4.0) kg/m^2^. The sample was 79.5% female (n=399/502) and 82.5% White (n=414/502). At 12 months, retention was 78.5% (n=394/502) and similar by group (SM+FB: 202/251, 80.5%; SM: 192/251, 76.5%; *P*=.28). There was significant percent weight loss from baseline in both groups (SM+FB: –2.12%, 95% CI –3.04% to –1.21%, *P*<.001; SM: –2.39%, 95% CI –3.32% to –1.47%; *P*<.001), but no difference between the groups (–0.27%; 95% CI –1.57% to 1.03%; *t* =–0.41; *P*=.68). Similarly, 26.3% (66/251) of the SM+FB group and 29.1% (73/251) of the SM group achieved ≥5% weight loss (chi-square value=0.49; *P*=.49). A 1% increase in FB messages opened was associated with a 0.10 greater percent weight loss at 12 months (b=–0.10; 95% CI –0.13 to –0.07; *t* =–5.90; *P*<.001). A 1% increase in FB messages opened was associated with 0.12 greater percentage of days adherent to the calorie goal per month (b=0.12; 95% CI 0.07-0.17; *F*=22.19; *P*<.001).

**Conclusions:**

There were no significant between-group differences in weight loss; however, the findings suggested that the use of commercially available digital SM tools with or without FB resulted in a clinically significant weight loss in over 25% of participants. Future studies need to test additional strategies that will promote greater engagement with digital tools.

**Trial Registration:**

Clinicaltrials.gov NCT03367936; https://clinicaltrials.gov/ct2/show/NCT03367936

## Introduction

Obesity is associated with several chronic diseases [1,2]. Obesity prevalence in the United States exceeds 42.4% and disproportionately affects racial and ethnic minority groups [3,4].

The gold standard for weight loss treatment is standard behavioral treatment (SBT), which includes reduced energy intake, increased energy expenditure, and in-person, group-based behavioral counseling plus feedback (FB) on self-monitoring (SM) from a trained interventionist [5-7]. However, SBT is difficult to implement on a large scale to reach populations most in need of treatment [8]. There is a critical need for more affordable, scalable, less burdensome, and efficacious treatments for weight loss [4].

The cornerstone of SBT is SM with interventionist FB [9-12]. A meta-regression demonstrated that SM use was the strongest predictor of efficacy in a weight loss intervention [13]. The highest efficacy was reported in a study in which SM was combined with another self-regulation technique [14], such as FB. Several studies have examined strategies to enhance sustained engagement in SM, including use of digital tools [5,11,12,15-20].

However, despite improvements in SM (eg, advancing from paper to digital tools), 2 issues persist: individuals still find SM burdensome [21] and SM adherence declines over time, which is associated with poorer weight loss outcomes [11,15,17-19,21-28]. Advances in mobile technology provide opportunities to enhance interventions targeting SM, expand their reach, and prevent decline in adherence. Delivering real-time FB to SM can reinforce behavior change [29] and partially replace in-person sessions [30,31]. The addition of wearable activity trackers [32] and smart scales [33] that synchronize data with a smartphone eliminates the need to manually record physical activity (PA) and weight, reducing burden and increasing adherence [19,34,35].

We previously examined the effect of providing FB to dietary SM and PA; however, the hardware and software used were rudimentary compared to today’s technology [16]. Despite these limitations, remotely delivered FB messages enhanced SM adherence and improved weight loss [29,36]. These results and significant mobile technology enhancements provided groundwork for the expanded algorithm and FB intervention used in this trial, SMARTER [23].

SMARTER was a 2-group randomized controlled trial that enrolled 502 adults with random assignment to either (1) SM alone (n=251) or (2) SM+FB (n=251) and examined the efficacy of the approaches. We examined short-term outcomes at 6 months with 86% retention that demonstrated that both groups lost a significant percent weight from baseline (SM+FB: –3.16%, *P*<.001; SM: –3.20%, *P*<.001) but found no significant between-group difference (*P*=.94) [37]. We hypothesized that the SM+FB group would show greater percent weight loss at 12 months compared to the SM group. For the SM+FB group, we also report the percent of FB messages opened and association with weight change and daily calorie goal adherence. We hypothesized that a greater number of FB messages that were opened would result in greater weight loss and adherence to the calorie goal.

## Methods

### Ethical Considerations

We published the study protocol and design previously [23]. The study was approved by the Institutional Review Board for Human Protection at the University of Pittsburgh (#19060112) and registered on ClinicalTrials.gov (NCT03367936). We informed all participants of screening procedures prior to obtaining consent and obtained in-person informed consent for the intervention study.

### Recruitment

Recruitment, conducted in the greater community surrounding Pittsburgh, PA, commenced in August 2018 and ended in March 2020. The intervention trial was completed in April 2021. Both online and in-person methods were used. Initially, interested individuals who were regular smartphone users completed surveys and a 5-day food diary in which they needed to record at least 700 calories of food intake per day to ensure that they could SM. Once deemed eligible, individuals had an in-person assessment to verify weight and height for BMI measures. Inclusion criteria were BMI between 27 and 43 kg/m^2^, completion of a 5-day electronic food diary, and ability to engage in moderate PA. Exclusion criteria were needing supervision of diet or PA, pregnancy, serious mental illness (eg, schizophrenia), alcohol abuse or eating disorder, and current weight loss treatment [23].

### Randomization

**Figure 1:**
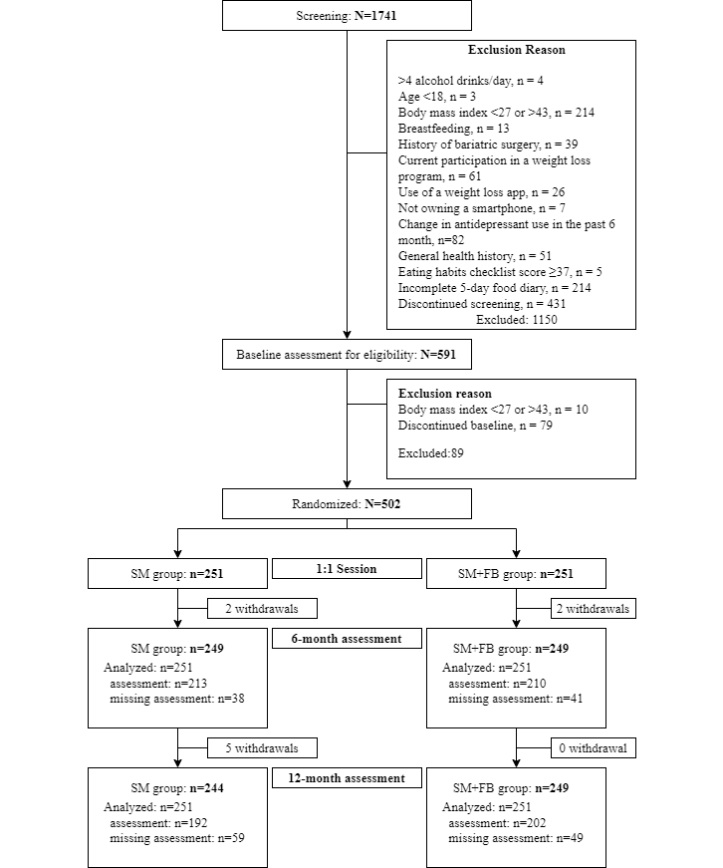
CONSORT (Consolidated Standards of Reporting Trials) diagram for the SMARTER trial.

After completing the intervention consent, research staff used a randomization software program to determine group assignment that was generated using minimization with stratification by gender (male or female) and race (Black or non-Black) with equal allocation to the 2 treatment conditions (please see the CONSORT [Consolidated Standards of Reporting Trials] diagram in Figure 1). All randomized participants were included in the final analyses. We used Wadden's conservative approach for imputing missing weight data assuming that there was a weight gain.

Key staff (BB, IL) who conducted the assessments were not blinded to the treatment assignment, whereas all other personnel and investigators, including the statisticians, (LEB, SMS, ZB, BP, JK, JC, MC, IWP, YW, and MBC) were blinded to assignment. Since participants were informed of both treatment conditions, they could not be blinded.

### Intervention

#### Behavioral Intervention

The intervention was grounded in behavioral change theory with an emphasis on Kanfer’s self-regulation theory that posits that SM is central to behavior change and includes FB tailored to the SM data. At baseline, all participants had a 90-minute, one-on-one, in-person intervention session with a dietitian on the core concepts of SBT followed by a demonstration of the Fitbit app to enter foods eaten for SM of diet, a Fitbit activity tracker to monitor PA, and a smart scale for daily self-weighing. Use of the investigator-developed SMARTER app, which was used only for random retrieval of FB messages from the message library and delivery of messages to the participant’s smartphone, was demonstrated to the SM+FB participants, so they could view the prompt icon for the FB messages and open the app to read the message. Participants used their own smartphones; the other SM devices (Fitbit activity tracker and commercially available smart scale) were provided by the study.

#### Dietary Intake

Participants used the Fitbit app to view food nutrient values, app-generated subtotals, and the daily intake summaries. The calorie goal was determined from baseline body weight (women: 1200 kcal for <200 lb or 1500 kcal ≥200 lb; men: 1500 kcal for <200 lb or 1800 kcal for ≥200 lb) and individualized as needed [23]. Fat gram goals approximated 25% of the calorie goal (eg, 33 or 42 grams per day for females).

#### Physical Activity

All participants monitored PA using a wrist-worn activity tracker, the Fitbit Charge 2, synced with their smartphone. Staff instructed participants to increase their PA gradually, primarily by walking, and to aim for 150 minutes per week by 12 weeks [38]. Once at goal, they were encouraged to add 10 minutes per week until they reached 300 minutes per week [23]. All aerobic activities counted toward PA goals. The Fitbit database stored total steps, sedentary minutes, and active minutes.

#### Weight

All participants were instructed to weigh daily on the study-provided smart scale that transmitted weight data to their smartphone and study database.

#### Feedback Messages

The FB algorithm was programmed on the study’s server and used real-time synced SM data to send the FB message up to 3 times per day. Individuals in the SM+FB group received up to 3 FB messages per day on their smartphone during waking hours tailored to the most recent SM data and addressing caloric as well as fat and added-sugar intake daily and PA every other day. Weekly weight FB was based on whether self-weighing occurred and the amount or rate of weight change. FB messages addressed 1 behavior at a time. The participant received a prompt that there was a new FB message on the smartphone. If the FB message was not opened within 1 hour of being sent, the SMARTER icon prompt and message disappeared; if the message was opened, the participant could save it for future review. More details on the FB messages and study infrastructure for message delivery are available elsewhere [23,37].

Engagement with SM tools was a crucial component of the intervention, as the algorithm used the SM data to determine an appropriate FB message. If the participant did not SM, FB messages were sent encouraging SM. After 2 weeks of missing SM data, staff sent an email query about technical issues and encouraged SM. Additional details on the algorithm and FB messages are published elsewhere [23]. The message library was changed at least monthly to avoid participant desensitization to FB [29]. Individuals in the SM group did not receive FB messages or staff emails. Further details of all intervention components and the algorithm driving the FB messages have been published elsewhere [23].

### Outcomes

#### Assessments

Both in-person and web-based assessments were used initially, and the in-person assessments were performed at the Clinical Research Center in the School of Nursing at the University of Pittsburgh. At in-person assessments, participants (in light clothing and bare feet) stood on a Tanita scale and body fat analyzer at baseline, 6 months, and 12 months. Percent lean and fat mass were also collected; however, after March 16, 2020 (ie, the beginning of the COVID-19 pandemic shutdown), we collected 12-month weight data remotely from the participants’ study-provided scales, which assessed only weight and percent fat mass. Staff contacted participants to instruct them to dress in clothing as in the baseline assessment and report their weight, which was also captured electronically [23]. At 12 months, of the 502 participants, 189 (37.7%) had in-person weights, 205 (40.8%) had remote weights, and 108 (21.5%) had missing weights. Smart scale weights recorded within 2 weeks of 6- or 12-month assessments were used for imputation of missing weights. If no weight was recorded by the smart scale, a 0.01 kg/day weight gain was assumed from last available scale weight value [39]. Cardiometabolic measures are reported in Multimedia Appendix 1 (Figures S1-S6). Participants were compensated for completing the 6- and 12-month assessments regardless of mode of conduct, in-person or remotely.

#### Feedback Messages

The monthly percentage of FB messages opened over the 12 months are expressed as the number of FB messages opened over the total number of FB messages sent in 30-day increments and multiplied by 100%.

#### Adherence to the Study-Defined Calorie Goal

The monthly percentage of days adherent to the calorie goal (defined as between 85% and 115% of daily calorie goal) among participants meeting the dietary SM goal (≥50% daily calorie goal) was calculated as follows:

Number of days meeting calorie goal per month / Number of days meeting dietary SM criteria per month × 100%

### Statistical Analysis

The planned total sample size for this randomized controlled trial was determined as 530 (265 per treatment group), allowing for a statistical power of 0.80 to detect effect sizes (standardized mean differences, *d*) as small as *d*=0.301 for the mean percent weight changes at 6 and 12 months between the SM-only and SM + FB groups when using linear mixed modeling with linear contrasts at a Bonferroni-adjusted significance level of *P*=.025 and for at most 20% attrition [23]. Due to the COVID-19 pandemic, recruitment was stopped in March 2020 with 502 randomized participants (251 per treatment arm). With this reduced sample size, slightly larger small-to-medium effect sizes of *d*=0.309 would still be detectable with 0.80 power at an adjusted significance level of *P*=.025, allowing for up to 20% attrition.

Continuous variables are summarized as mean and SD, and descriptive statistics for categorical variables are reported as counts with percentages. Appropriate group comparative analyses were performed on participant descriptors and outcome variables at baseline by randomized treatment assignment [37]. The effect of treatment assignment on percent weight change over 12 months was examined using linear mixed modeling following intention to treat. Models included a random intercept and unstructured variance-covariance matrix for the repeated assessments, supported by Akaike information and Bayesian information criteria. The base model included fixed effects for time (baseline vs 6 months and 12 months), group (SM+FB vs SM alone), and group by time interaction. Sensitivity analyses were performed among completers only and using inverse probability weighting for dropout by 12 months with age, race, and baseline as predictors.

The effect of the percentage of FB messages opened on percent weight change from baseline to 12 months for the SM+FB group was analyzed using univariate linear regression. Additionally, the associations of monthly percentages of days adherent to the calorie goal with treatment assignment and monthly percentages of FB messages opened were analyzed using separate linear mixed models with random intercept and slope for the total sample and for the SM+FB group, respectively. We conducted sensitivity analyses on the treatment effects on monthly percentages of days adherent to the calorie goal over 12 months in the total sample and on the associations between monthly percentages of FB messages opened and monthly percentages of days adherent to the calorie goal in the SM+FB group for the varying monthly percentage of days with sufficient dietary SM data (data not shown). Here, we report the results using days with ≥50% of the calorie goal recorded or ≥15 of 30 days with sufficient dietary SM data.

Model assessment (ie, residual analyses with influence diagnostics) was performed for each fitted model; sensitivity analyses were conducted for outlying or influential observations and to explore the effect of the COVID-19 pandemic on the efficacy of treatment assignment on percent weight change (data not shown). All analyses were performed using SAS version 9.4 (SAS Institute).

## Results

### Baseline Characteristics

Most participants were White (414/502, 82.5%), female (399/502, 79.5%) and on average 45.0 (SD 14.4) years old. Sociodemographic, clinical, and psychosocial characteristics, as well as primary outcome measurements at baseline, were similar between the treatment groups [37].

### Retention

At 12 months, the overall retention was 78.5% (394/502). Retention was similar by treatment condition, with SM+FB at 80.5% (202/251) and SM at 76.5% (192/251; *X*^2^=1.18; *P*=.28).

### Percent and Absolute Weight Change

**Figure 2:**
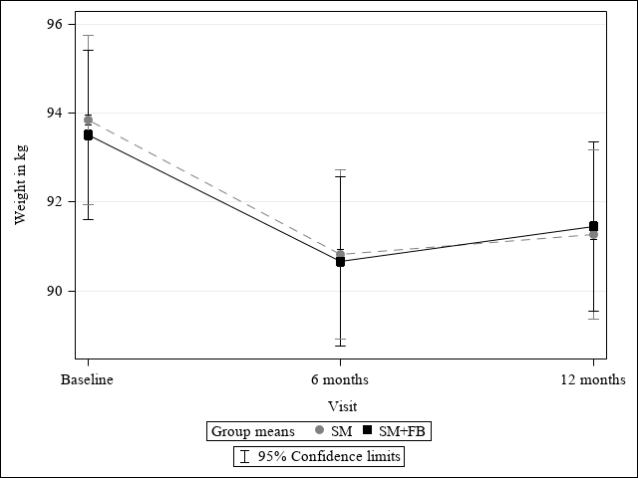
Effect of treatment assignment on weight change.

**Figure 3:**
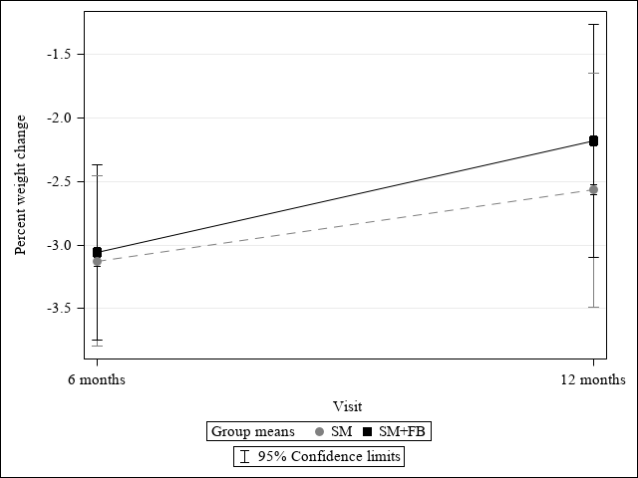
Effect of treatment assignment on percent weight change.

Figures 2 and 3 illustrate the results from linear mixed modeling for the effect of treatment assignment on weight loss and percentage of weight loss over 12 months. On average, both groups had statistically significant weight loss over 12 months (b_6 months_=–2.94, 95% CI –3.70 to –2.19; b_12 months_=–2.34, 95% CI –3.10 to –1.59; *F*= 61.46; *P*<.001). The trajectory of weight change over time was similar between groups (b_group × 6 months_=0.09, 95% CI –0.97 to –1.16; b_group × 12 months_=0.36, 95% CI –0.70 to –1.43; *F*=0.24; *P*=.79), and there were no significant overall treatment effects on weight change (b_group_=–0.32; 95% CI –3.04 to 2.40; *F*=0.02; *P*=.*90*). There was a significant percent weight loss from baseline in both groups (SM+FB: –2.12%, 95% CI –3.04% to –1.21%, *P*<.001; SM: –2.39%, 95% CI –3.32% to –1.47%, *P*<.001), but no difference between the groups (–0.27%; 95% CI –1.57% to 1.03%; *t*=–0.41; *P*=.68). The percentages of participants who lost ≥5% weight from baseline to 12 months were similar between the SM+FB (66/251, 26.3%) and SM (73/251, 29.1%) arms (*Χ*^2^=0.49; *P*=.49). Based on sensitivity analyses using inverse probability weighting, there was no significant difference in percent weight change at 12 months in the SM+FB arm (mean –3.57%, SD 20.16) and the SM arm (mean –3.53%, SD 19.94; *t*=0.07; *P*=.95). Additional analyses among completers showed no significant treatment effects on percent weight change at 12 months (SM+FB: mean –3.54%, SD 7.16; SM mean: –3.58%, SD 7.06; *t*= –0.07; *P*=.95). For the total sample, mean weight change in kilograms at 12 months was mean –2.16 kg (SD 0.27). There was no significant difference in weight change in kilograms at 12 months in the SM+FB arm (mean –1.98 kg, SD 0.38) or the SM arm (mean –2.34, SD 0.38; 2-sample *t* test=0.67; *P*=.50).

Analyses without outliers had results similar to the full-sample results. There were no significant effects for the COVID-19 pandemic on the relationship of treatment with weight change over time.

### Feedback Messages Opened

**Figure 4:**
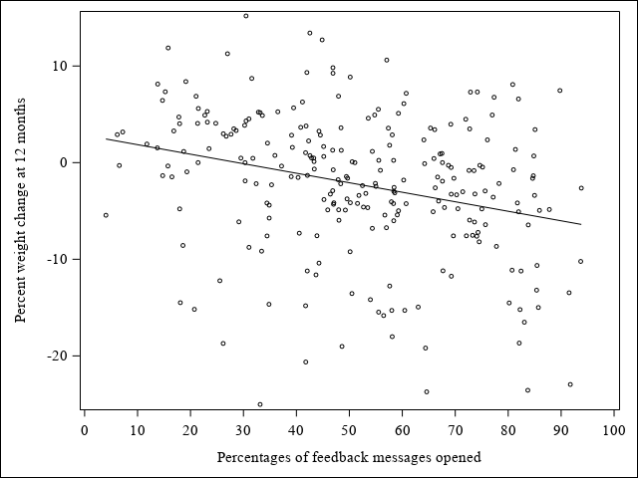
The associations between percentages of feedback messages opened and percent weight change at 12 months.

In the SM+FB arm, the median percentage of FB messages opened from baseline to 12 months was 42.19% (461/1026; q1: 234/597, 39.20%; q3: 728/1095, 66.48%) and ranged from 1.28% (14/78) to 93.70% (1026/1095). Figure 4 displays the association of the percentage of FB messages opened and percent weight change at 12 months. A 1% increase in FB messages opened was associated with a 0.10 greater percent weight loss at 12 months (b=–0.10; 95% CI –0.13 to –0.07; *t*=–5.90; *P*<.001).

### Percentage of Days Adherent to the Calorie Goal

**Figure 5:**
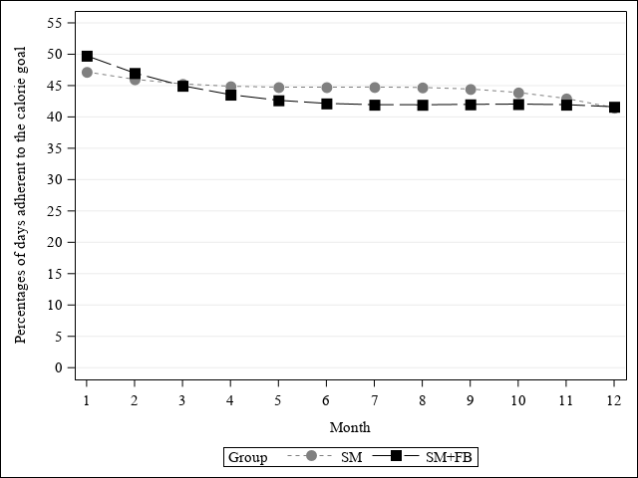
Effect of treatment assignment on percentage of days adherent to the calorie goal over 12 months.

Figure 5 illustrates the change in monthly percentage of days adherent to the calorie goal by treatment group over 12 months using the criteria of recording ≥50% of the calorie goal 15 days per month. The monthly percentage of days adherent to the calorie goal declined nonlinearly in both groups. Overall, the percentage of days adherent to the calorie goal was greater in the SM+FB group than in the SM group (b_group_=4.43; 95% CI 0.41-8.45; *F*=4.67; *P*=.03). The rate of decline in percentage of days adherent to the calorie goal was slower in the SM+FB arm than the SM arm over 12 months (b_group_ _× time-linear_=–1.98, 95% CI –3.03 to –0.93, *F*=13.71, *P*<.001; b_group_ _× time-quadratic_=0.14, 95% CI 0.06-0.22, *F*=11.04, *P*<.001).

**Figure 6:**
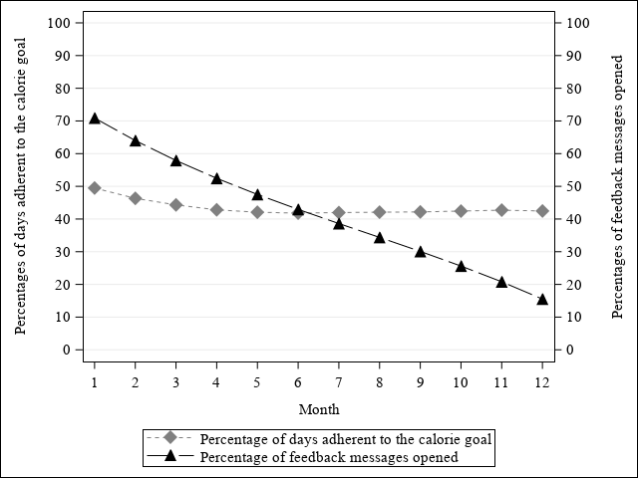
Change in monthly percentages of feedback messages opened and days adherent to the calorie goal in SM+FB over 12 months.

**Figure 7:**
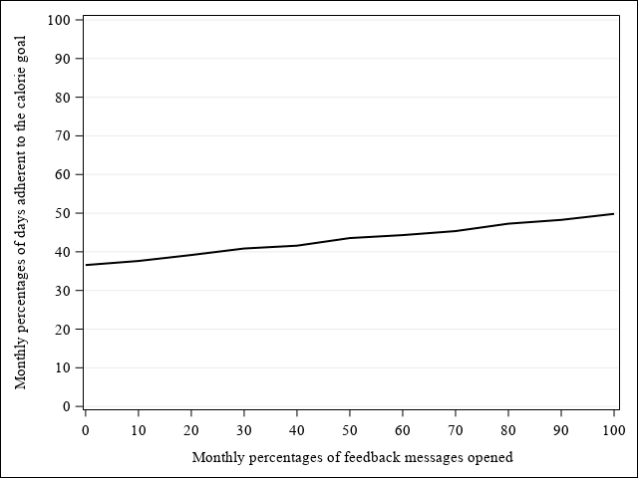
The associations between monthly percentages of feedback messages opened and days adherent to the calorie goal in SM+FB over 12 months.

Figure 6 illustrates the change in monthly percentages of FB messages opened and monthly percentages of days adherent to the calorie goal over 12 months and their association in the SM+FB group, respectively. In general, monthly percentages of FB messages opened (b_time-linear_=–8.34, 95% CI –9.91 to –6.78, *F*=110.26, *P*<.001; b_time-quadratic_=0.54, 95% CI 0.27-0.81, *F*=15.07, *P*<.001; b_time-cubic_=–0.02, 95% CI –0.04 to –0.009, *F*=10.63, *P*=.001) and monthly percentages of days adherent to the calorie goal declined nonlinearly over 12 months (b_time-linear_=–3.37, 95% CI –5.29 to –1.45, *F*=11.93, *P*=.001; b_time-quadratic_=0.44, 95% CI 0.10-0.79, *F*=6.26, *P*=.01; b_time-cubic_=–0.02, 95% CI –0.04 to 0.001, *F*=3.62, *P*=.06), with a greater percentage of FB messages opened being associated with higher adherence to the calorie goal as shown in Figure 7 (b_FB_=0.12; 95% CI 0.07 to 0.17; *F*=22.19; *P*<.001). There was no significant interaction between the percentage of FB messages opened and the polynomial time effects.

## Discussion

### Principal Results

We conducted a trial of a scalable, remotely delivered, behavioral weight loss intervention and tested the efficacy of a custom-developed, theoretically based, smartphone app that provided real-time FB remotely to reinforce diet, PA, and self-weighing behaviors. We observed a small but significant percent weight change from baseline to 12 months with no significant difference between the groups, suggesting the FB provided no additional benefit beyond SM. Our findings suggest that it is feasible to deliver a 12-month, remotely delivered intervention for weight loss to a large sample even with COVID-19 pandemic restrictions. The percent weight losses observed (equivalent to an absolute weight change of 2.0-2.5 kg) were less than what is usually reported in studies of in-person group counseling or online coaching [40-42] but not different from other small trials that tested digital interventions with limited human interaction [43]. A systematic review of eHealth interventions reported similar weight losses (1.4 to 2.7 kg) [44] at postintervention [45].

In this paper, we report initial app engagement findings, specifically the number of times participants opened the app to SM or read FB messages. Our study demonstrated that without personal interaction, engagement declined to the point that it interfered with the intervention delivery. Although the FB message system worked in theory, the declining adherence to SM created a lack of data for the algorithm to select a FB message. Sending an email message after 2 weeks without evident SM might have been too long to wait to prompt re-engagement, and a phone call might have been more effective; however, we were trying to increase the study scalability. The COVID-19 shutdown ended all in-person interactions including assessments, which might have affected engagement.

We measured engagement by the percentage of FB messages opened, which was reflective of dietary SM adherence. Overall, approximately 40% (461/1026, 42.19%; IQR 45.30) of the FB messages were opened with a very wide range of 1.28% (14/78) to 93.70% (14/78) in our study. Although this is less than ideal, engagement was equal (or greater) than the 41% to 60% rate of engagement that others have reported [46-48] in mobile health studies. A recent literature review emphasized the challenge of initial and sustained engagement in mobile health studies [49]. Considering the completely remote intervention with minimal to no in-person contact, our findings are encouraging but indicate a continued need to improve sustained engagement in SM [50].

### Comparison With Prior Work

There was greater adherence to the calorie goal in the SM+FB group compared to the SM group, and a greater number of FB messages opened was associated with a greater percent weight loss in the SM+FB group, suggesting that when messages were opened, the FB messages reinforced behavior changes related to food selection. These findings are similar to those in the SMART trial which showed that the personal data assistant + FB group had better adherence to the dietary goals and was the only 1 of the 3 study groups that had a significant within-group weight loss over 24-months. In SMART, adherence to SM strongly predicted weight loss at all time points [17,29,49].

The study algorithm and FB message library for SMARTER were significantly expanded from the earlier SMART trial; however, these improvements did not compensate for the absence of the 16 in-person group sessions that were part of the SMART trial, suggesting that some form of interpersonal interaction may be needed to augment mobile health interventions. Recent studies have reported similar findings [40,42]. For example, Thomas et al [40] demonstrated that providing a monthly in-person weigh-in to accompany 5-minute skills training videos achieved weight losses comparable to the gold standard of frequent in-person group sessions over the 18-month trial. Similarly, Amagai et al’s [49] literature review suggested that coaching to provide social support is an important strategy to improve engagement.

The comparison group in SMARTER received the same treatment components (ie, a one-to-one, in-person intervention session at baseline and digital tools for SM) and achieved very similar weight loss without receiving any prompts or reminders to SM. The intent of this comparative intervention was to determine the effectiveness of the approach that thousands of individuals are using by purchasing apps and tracking devices. These results suggest that some individuals who receive individual guidance at baseline with goals for diet and PA and encouragement to SM can achieve a clinically significant weight loss under their own direction.

Both groups used the Fitbit Charge 2 for tracking diet and PA. The Fitbit provided graphical FB on dietary intake (total calories consumed and “burned”) and a weekly summary of PA; thus, the SM-only group received some automatic FB if they were syncing their device to their phone. Although this FB could be reinforcing for some individuals, it lacked the personalized component that the SMARTER FB provided since the SMARTER messages were tailored to SM data entered at that time, were positive in tone, and often provided suggestions. However, due to the lower-than-expected engagement with SM, many individuals did not open (and therefore receive) enough messages.

A recent pilot study that used a 2 × 2 factorial design provides some insights into FB (counselor-crafted vs pre-scripted [51]) and group sessions (yes or no). Participants in the group sessions were more engaged in SM and lost more weight than did those not offered group sessions; however, the group that received pre-scripted, modular FB had significantly greater weight loss than did the group that received the counselor-crafted FB while there was no consistent difference in their treatment engagement. It is not known why the group that received briefer FB lost more weight; the longer FB sent weekly was possibly perceived as burdensome. The authors and other researchers suggested that the mechanisms underlying FB are poorly defined and that the amount, timing, frequency, and framing are just a few of the dimensions that need to be further studied [52-54].

Several recent weight loss intervention studies have examined an array of digital strategies to enhance adherence to SM while reducing components of the gold standard SBT; however, several had small samples, conducted brief interventions, and had small weight losses [26,43,55]. Despite these limitations, results showed promise for further study of approaches to enhance SM adherence (eg, counseling phone calls [26] or weekly emails with structured lessons [43]). The cumulative evidence makes it difficult to determine which intervention components can be most effective in producing clinically meaningful weight loss. Specifically, it is difficult to ascertain how much of the human interventionist component can be replaced to make weight loss treatments scalable to a broader reach and lower operational costs. This critical gap in the evidence needs to be addressed in future studies, so we can broaden our reach to the millions who need weight loss treatment, particularly those who do not have access to existing clinical and commercial weight loss programs.

### Study Strengths

There are several strengths to our study: a large sample size, a rigorous randomized design with a comparable control group, a retention rate higher than that reported by shorter and similar studies [56], use of validated measures, defined adherence metrics, and an objective measure of FB messages opened. The theory-based intervention was expanded from a previously tested and efficacious FB system. Additional strengths include using an extensive remote screening system and, born of necessity due to the COVID pandemic, pivoting to a remotely delivered, objective assessment protocol with minimal data loss.

### Study Limitations

Limitations include recruitment of fewer males and minorities than targeted, which limits generalizability. The retention was slightly lower than the targeted 80%. The metric of FB messages opened does not necessarily equate to the actual number of FB messages read.

### Conclusions

The SMARTER trial delivered customized, real-time FB to participants based on SM data and capitalized on the use of available digital technology to provide personal weight management support without ongoing human counseling. This approach is scalable, as it reduces cost and participant burden while increasing reach to those without access to SBT or who do not wish to participate in an in-person program. We hypothesized that participants in the SM+FB group would have greater weight loss than those in the SM group at 12 months; however, weight loss outcomes were similar. Our results suggest that the addition of FB to SM did not make a significant between-group difference in weight loss outcomes; however, those who remained engaged and opened more FB messages had better calorie goal adherence and weight outcomes. Moreover, one-fourth and almost one-third of each group achieved clinically significant weight loss, suggesting that for a portion of participants, the SM component of the intervention was efficacious.

Considering the unrelenting prevalence of obesity, there is a critical need for scalable interventions that can reach those most at risk and with the least resources. The evidence supports standalone, scalable digital interventions, yet the crucial challenge is the development of digital tools that will keep users engaged long enough to see positive, sustainable outcomes. In advancing the digital aspects, we also need to identify the most efficacious personal interaction components that best augment and support sustained SM and lifestyle change. Obesity is a complex, multifactorial, chronic condition that requires ongoing support and an array of treatment options that will accommodate for the diverse needs of those seeking treatment.

## Appendix

Multimedia Appendix 1 Cardiometabolic measures.

Multimedia Appendix 2 CONSORT-eHEALTH checklist (V 1.6.2).

## Abbreviations

CONSORT: Consolidated Standards of Reporting Trials
FB: feedback
NIH: National Institutes of Health
PA: physical activity
SBT: standard behavioral treatment
SM: self-monitoring
