# Supplementary material for: The Effect of Tailored, Daily, Smartphone Feedback to Lifestyle Self-Monitoring on Weight Loss at 12 Months: the SMARTER Randomized Clinical Trial
Source: J Med Internet Res. 2022 Jul 5;24(7):e38243. doi: 10.2196/38243 (PMC9297147; doi:10.2196/38243)

**Multimedia Appendix 1: Cardiometabolic Outcome Measures**

Figure S1. Treatment Effects on Body Mass Index over 12 Months in the SMARTER Trial Participants

Figure S2. Treatment Effects on Percent Body Fat over 12 Months in the SMARTER Trial Participants

Figure S3. Treatment Effects on Waist Circumference over 12 Months among Males in the SMARTER Trial Participants

Figure S4. Treatment Effects on Waist Circumference over 12 Months among Females in the SMARTER Trial Participants

Figure S5. Treatment Effects on Systolic Blood Pressure over 12 Months in the SMARTER Trial Participants

Figure S6. Treatment Effects on Diastolic Blood Pressure over 12 Months in the SMARTER Trial Participants

**Supplement Materials: Cardiometabolic Outcome Measures**

For in-person assessments, waist circumference was measured twice following an established protocol^1^ and systolic and diastolic blood pressure (BP) assessments followed the standard American Heart Association guidelines.^2^ BP and waist circumference were not measured at remote assessments. Due to the COVID-19 restrictions, in-person assessments were not conducted after March 17, 2020. At 12 months, 189 (37.7%) participants had in-person assessments, 205 (40.8%) had remote assessments, and 108 (21.5%) were missing assessment data.

Additional analyses were performed for secondary outcomes such as BMI, percent body fat, waist circumference for males and females separately, and systolic and diastolic blood pressure. The residual diagnostics for levels of subjects were performed for each model and were re-fit after removal of extreme observations using Cook’s D and PRESS statistics.

Overall, there was no treatment effect on change in BMI over 12 months (β _SM+FB_ = 0.006; 95% CI -0.70 to 0.72; *F* _group_ =0.36_;_ *P*=.551). However, there was a time effect which suggests that both groups had a significant change in BMI (β _6 months_ = -1.28; 95% CI -1.56 to -1.01; β _12 months_ = -1.41; 95% CI -1.76 to -1.06; *F* =86.32_;_ *P*=<.0001). Most importantly, the trajectory of change in BMI was not significantly different between groups (β _SM+FB at 6 months_ = 0.16; 95% CI -0.23 to 0.56; β _SM+FB at 12 months_ = 0.46; 95% CI -0.04 to 0.95; *F* =1.67_;_ *P*=.189) (e*Figure S1*).

There was no treatment effect on change in % body fat over 12 months (β _SM+FB_ = 0.43; 95% CI -0.83 to 1.68; *F* = 2.04_,_ *P*=.154). However, there was a time effect which suggests that both groups had a significant change in % body fat (β _6 months_ = -1.87; 95% CI -2.46 to -1.28; β _12 months_ = -2.01; 95% CI -2.76 to -1.26; *F* =34.16_,_ *P*=<.0001). Most importantly, the trajectory of change in % body fat was not significantly different between groups (β _SM+FB at 6 months_ = 0.46; 95% CI -0.38 to 1.29; β _SM+FB at 12 months_ = 0.96; 95% CI -0.11 to 2.02; *F* =1.66_;_ *P*=.191) (e*Figure S2*).

Among male participants, there was no treatment effect on change in waist circumference over time (β _SM+FB_ = -3.40; 95% CI -8.22 to 1.42; *F*=0.09_,_ *P*=.760). However, there was a time effect which suggests that both groups had a significant decrease in waist circumference (β _6 months_ = -5.65; 95% CI -7.77 to -3.52; β _12 months_ = -7.41; 95% CI -10.12 to -4.71; *F* =22.44_,_ *P*=<.0001). Most importantly, the trajectory of change in waist circumference over time was significantly different between groups (β _SM+FB at_ _6 months_ = 2.07; 95% CI -0.87 to 5.02; β _SM+FB at_ _12 months_ = 5.91; 95% CI 2.13 to 9.68; *F*=4.84_,_ *P*=.010). Specifically, from baseline to 12 months, the SM group (mean ± se: -7.41 ± 1.37) had significantly larger decrease in waist circumference than the SM+FB group (mean ± se: -1.51 ± 1.33, *P*=.003) (e*Figure S3*).

Among females, there was no treatment effect on change in waist circumference over time (β _SM+FB_ = 0.55; 95% CI -1.70 to 2.81; *F* =0.15_,_ *P*=.695). However, there was a time effect which suggests that both groups had a significant decrease in waist circumference (β _6 months_ = -2.59; 95% CI -3.67 to -1.52; β _12 months_ = -3.30; 95% CI 4.66 to 1.93; *F* =34.27_,_ *P*=<.0001). Importantly, the trajectory of change in waist circumference was not significantly different between groups over time (β _SM+FB at_ _6 months_ = -0.35; 95% CI -1.88 to 1.19; β _SM+FB at_ _12 months_ = 0.02; 95% CI -1.93 to 1.96; *F* _group_ _× time_=0.12_,_ *P*=.889) (e*Figure S4*).

There was no treatment effect and time effect on change in SBP over time (respectively - *F* _treatment_ = 0.48_,_ *P*=.489; *F* _time_ =0.80_,_ *P*=.450). Importantly, the effect of treatment assignment on SBP did not differ by group over time (*F* _treatment_ _× time_=1.14, *P*=.321) (*Figure S5*). Overall, there was no treatment effect and time effect on change in DBP over time (respectively - *F* _treatment_ =0.20_,_ *P*=.652; *F* _time_ =0.89_,_ *P*=.411). The effect of treatment assignment on DBP did not vary by group (*F* _treatment_ _× time_=0.53_,_ *P*=.587) (e*Figure S6*).

References

1. Gulick II Instruction Manual. In. Gays Mills, WI 54631: Country Technology, Inc; 2007.

2. Flack JM, Adekola B. Blood pressure and the new ACC/AHA hypertension guidelines. *Trends Cardiovasc Med.* 2020;30(3):160-164.

**Figure 1. Treatment Effects on Body Mass Index over 12 Months in the SMARTER Trial Participants**


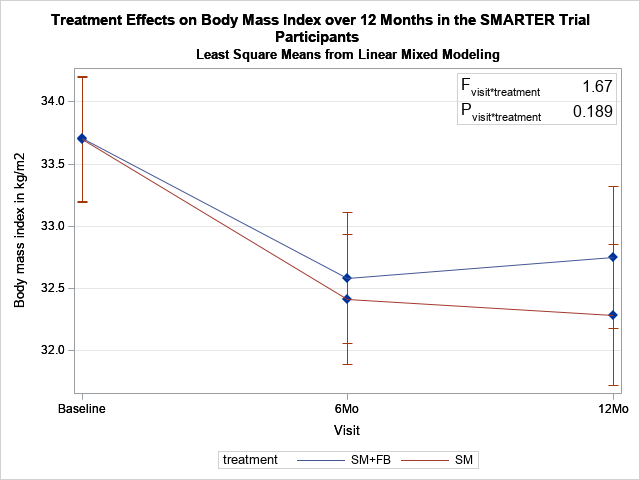


**Figure 2. Treatment Effects on Percent Body Fat over 12 Months in the SMARTER Trial Participants**


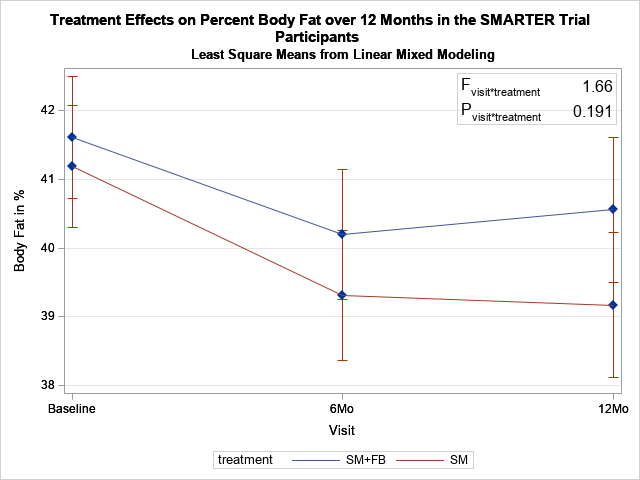


**Figure 3. Treatment Effects on Waist Circumference over 12 Months among Males in the SMARTER Trial Participants**


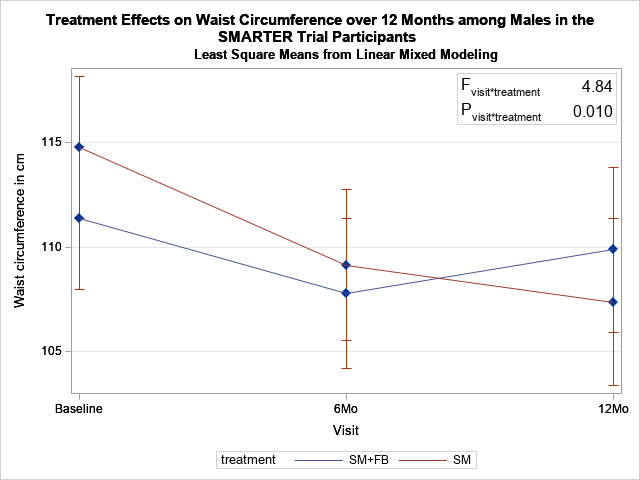


**Figure 4. Treatment Effects on Waist Circumference over 12 Months among Females in the SMARTER Trial Participants**


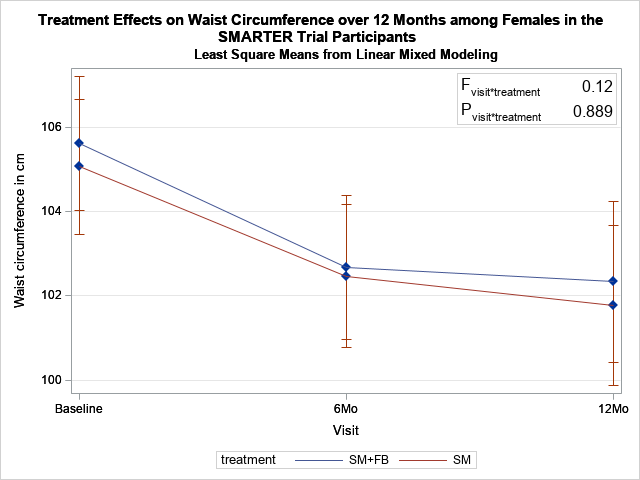


**Figure 5. Treatment Effects on Systolic Blood Pressure over 12 Months in the SMARTER Trial Participants**


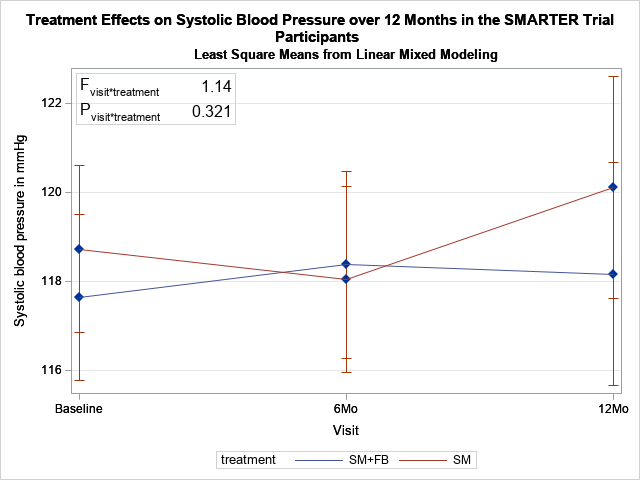


**Figure 6. Treatment Effects on Diastolic Blood Pressure over 12 Months in the SMARTER Trial Participants**


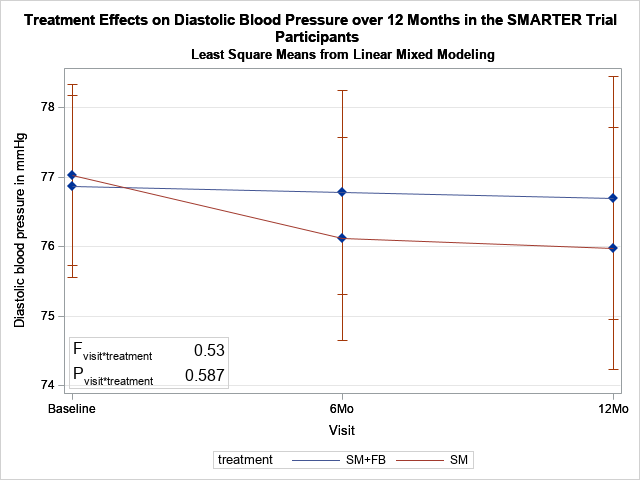

Supplement: Multimedia Appendix 1 [file jmir_v24i7e38243_app1.docx]
