# Supplementary material for: The Effect of Tailored, Daily, Smartphone Feedback to Lifestyle Self-Monitoring on Weight Loss at 12 Months: the SMARTER Randomized Clinical Trial
Source: J Med Internet Res. 2022 Jul 5;24(7):e38243. doi: 10.2196/38243 (PMC9297147; doi:10.2196/38243)
Supplement: Multimedia Appendix 2 [file jmir_v24i7e38243_app2.pdf]

CONSORT-EHEALTH Checklist V1.6.2 Report	Manuscript Number	38243

(based on CONSORT-EHEALTH V1.6), available at [http://tinyurl.com/consort-ehealth-v1-6].

Date completed

6/17/2022 23:45:09

by

Lora E Burke

The Effect of Tailored, Daily Smartphone Feedback to Lifestyle Self-Monitoring on Weight Loss at 12 Months: The SMARTER Randomized Clinical Trial

TITLE

1a-i) Identify the mode of delivery in the title

Remotely delivered feedback messages. Individuals in SM+FB group received up to three FB messages per day during waking hours tailored to SM data and addressing caloric, fat and added-sugar intake daily and PA every other day. Weekly weight FB was based on whether self-weighing occurred and the amount/rate of weight change. FB messages addressed one behavior at a time. If the FB message on the smartphone was not opened within one hour of being sent, it disappeared; if the message was opened, the participant

1a-ii) Non-web-based components or important co-interventions in title

1a-iii) Primary condition or target group in the title

Behavioral intervention. The intervention is grounded in behavioral change theory with an emphasis on Kanfer's self-regulation theory that posits that self-monitoring is central to behavior change and includes feedback tailored to the self-monitoring data. At baseline, all participants had a 90-minute 1:1 in-person intervention session with a dietitian on core concepts of SBT followed by a demonstration of the Fitbit app to SM diet, a Fitbit activity tracker to monitor PA, and a smart scale for daily self-weighing. Use of the investigator-developed SMARTER app, which was used only for random retrieval of feedback messages from the message library and delivery of message to the participant's smartphone, was demonstrated to the SM+FB participants so they could view the prompt icon for the feedback messages and open the app to read the message. Participants used their own smartphones; the other self-monitoring devices (Fitbit activity tracker and commercially-available smart scale) were provided by the study.

ABSTRACT

1b-i) Key features/functionalities/components of the intervention and comparator in the METHODS section of the ABSTRACT

"All participants received a 90-minute 1:1 in-person behavioral weight loss counseling session addressing behavioral strategies, establishing participant's dietary and PA goals, and instructing on use of PA tracker (Fitbit Charge 2™), smart scale, and diet SM app. Only SM+FB participants had access to an investigator-developed smartphone app that read SM data, selected tailored messages sent to smartphone up to 3 times daily. The SM only participants did not receive any tailored feedback based on SM data. "

1b-ii) Level of human involvement in the METHODS section of the ABSTRACT

As above, the human involvement was described as 1:1 personal intervention with a dietitian for 90 minutes at baseline.

1b-iii) Open vs. closed, web-based (self-assessment) vs. face-to-face assessments in the METHODS section of the ABSTRACT

"The primary outcome was percent weight change from baseline to 12 months. Secondary outcomes included engagement with digital tools (e.g., monthly percentage of feedback messages opened and monthly percentage of days adherent to calorie goal)."
Due to the COVID-19 shutdown, in-person assessments were not conducted and measurements were collected from the weight scale provided to the study participant. This is described in detail in the manuscript.

1b-iv) RESULTS section in abstract must contain use data

"Participants (N=502) were on average 45.0 (SD 14.4) years old with BMI of 33.7 (SD 4.0) kg/m2. The sample was 79.5% female (n=399) and 82.5% white (n=414). At 12 months, retention was 78.5% (n=394) and similar by group (SM+FB: 80.5%, SM: 76.5%, P=.277) ."

1b-v) CONCLUSIONS/DISCUSSION in abstract for negative trials

There were no significant between group differences in weight loss; however, results suggest that the use of commercially available digital self-monitoring tools with or without feedback can result in a clinically significant weight loss in over 25% of participants. Future studies need to test additional strategies that will promote greater engagement with digital tools.

INTRODUCTION

2a-i) Problem and the type of system/solution

"Obesity is associated with several chronic diseases.[1, 2] Obesity prevalence in the United States exceeds 42.4% and disproportionately affects racial and ethnic minority groups.[3, 4]
The "gold standard" for weight loss treatment is standard behavioral treatment (SBT), which includes reduced energy intake, increased energy expenditure, and in-person, group-based behavioral counseling plus feedback (FB) on self-monitoring (SM) from a trained interventionist.[5-7] However, SBT is difficult to implement on a large scale to reach populations most in need of treatment.[8] The need for more affordable, scalable, less burdensome, and efficacious treatments for weight loss is crucial.[4]
The cornerstone of SBT is SM with interventionist FB.[9-12] A meta-regression demonstrated that SM use was the strongest predictor of efficacy in a weight loss intervention.[13] The highest efficacy was observed when SM was combined with another self-regulation technique,[14] such as FB. Several studies have examined strategies to enhance sustained engagement in SM, including use of digital tools.[5, 12, 15-21]
However, despite improvements in SM (e.g., advancing from paper to digital tools), two issues persist: individuals still find SM burdensome [22] and SM adherence declines over time, which is associated with poorer weight loss outcomes.[11, 16, 18-20, 23-30] Advances in mobile technology provide opportunities to enhance interventions targeting SM, expand their reach, and prevent decline in adherence. Delivering real-time FB to SM can reinforce behavior change [31] and partially replace in-person sessions [32, 33]. The addition of wearable activity trackers [34] and smart scales [35] that synchronize data with a smartphone eliminates the need to manually record physical activity (PA) and weight, reducing burden and increasing adherence [20, 36, 37] "

2a-ii) Scientific background, rationale: What is known about the (type of) system

"Several studies have examined strategies to enhance sustained engagement in SM, including use of digital tools.[5, 12, 15-21] However, despite improvements in SM (e.g., advancing from paper to digital tools), two issues persist: individuals still find SM burdensome [22] and SM adherence declines over time, which is associated with poorer weight loss outcomes.[11, 16, 18-20, 23-30] Advances in mobile technology provide opportunities to enhance interventions targeting SM, expand their reach, and prevent decline in adherence. Delivering real-time FB to SM can reinforce behavior change [31] and partially replace in-person sessions [32, 33]. The addition of wearable activity trackers [34] and smart scales [35] that synchronize data with a smartphone eliminates the need to manually record physical activity (PA) and weight, reducing burden and increasing adherence [20, 36, 37]."

We previously examined the effect of providing feedback to dietary SM and PA; however, the hardware and software used was rudimentary compared to today's technology.[17] Despite those limitations, remotely delivered FB messages enhanced SM adherence and improved weight loss.[31, 38] Those results and significant mobile technology enhancements provided groundwork for the expanded algorithm and FB intervention used in the current trial, SMARTER.[25]

Does your paper address CONSORT subitem 2b?

We previously examined the effect of providing feedback to dietary SM and PA; however, the hardware and software used was rudimentary compared to today's technology.[17] Despite those limitations, remotely delivered FB messages enhanced SM adherence and improved weight loss.[31, 38] Those results and significant mobile technology enhancements provided groundwork for the expanded algorithm and FB intervention used in the current trial, SMARTER.[25]

METHODS

3a) CONSORT: Description of trial design (such as parallel, factorial) including allocation ratio

SMARTER was a 2-group randomized controlled trial that enrolled 502 adults with random assignment to either 1) SM alone (n=251) or 2) SM+FB (n=251) and examined the efficacy of the approaches.

3b) CONSORT: Important changes to methods after trial commencement (such as eligibility criteria), with reasons

Percent lean and fat mass were also collected; however, after March 16, 2020 (i.e., COVID-19 pandemic shutdown start) we collected 12-month weight data remotely from the participant's study-provided scale, which assessed only weight and percent fat mass. Staff contacted participants to instruct them to dress in clothing like the baseline assessment and report their weight, which was also captured electronically.[25] At 12 months,189 (37.7%) participants had in-person weights, 205 (40.8%) had remote weights, and 108 (21.5%) were missing weights. Smart scale weights recorded within 2 weeks of 6- or 12-month assessments were used for imputation of missing weights. If no weight was recorded by the smart scale, a 0.01 kg/day weight gain was assumed from last available scale weight value.[43]

3b-i) Bug fixes, Downtimes, Content Changes

During the intervention, we did encounter minor bugs or issues on the app that we addressed in timely manner therefore minimized the impact on the participants and overall intervention. The app was designed to support auto update as well.

4a) CONSORT: Eligibility criteria for participants

" interested individuals who were regular smartphone users completed surveys and a 5-day food diary in which they needed to record at least 700 calories of food intake/day to ensure that they could self-monitor. Once deemed eligible, individuals had an in-person assessment to verify weight and height for body mass index measures. Inclusion criteria were body mass index (BMI) between 27 and 43 kg/m2, completion of a 5-day electronic food diary, and ability to engage in moderate PA. Exclusion criteria were needing supervision of diet or PA, pregnancy, serious mental illness (e.g., schizophrenia), alcohol abuse or eating disorder, and current weight loss treatment.[25]"

4a-i) Computer / Internet literacy

Individuals had to be regular smartphone users to be eligible for the study which means that they carried the phones with them regularly and kept the phone charged. We also did a run-in 5-day self-monitoring of dietary intake using a phone app as part of eligibility criteria screening.		
**4a-ii) Open vs. closed, web-based vs. face-to-face assessments:**		
"Both online and in-person methods were used to recruit individuals." All surveys for screening were conducted online. After initial eligibility was determined, individuals came into the center for the measurement of height and weight to determine body mass index and complete the first 24-hour dietary recall under supervision.		
**4a-iii) Information giving during recruitment**		
This paper addresses this briefly as the details are in the previously published methods paper. The intervention consent form was uploaded in the multimedia materials section.		
**4b) CONSORT: Settings and locations where the data were collected**		
"We used a combination of online and in-person recruitment/screening procedures. Initial screening of eligibility criteria was conducted nline with online consent form to collect these data. For the verification of weight/height for the calculation of body mass index (BMI) we conducted in-person assessment.:		
**4b-i) Report if outcomes were (self-)assessed through online questionnaires**		
As indicated previously, yes, all surveys were completed online. "We used a combination of online and in-person recruitment/screening procedures. Initial screening of eligibility criteria was conducted online with online consent form to collect these data. For the verification of weight/height for the calculation of body mass index (BMI) we conducted in-person assessment."		
**4b-ii) Report how institutional affiliations are displayed**		
The University of Pittsburgh letterhead is on the first page of each consent form.		
**5) CONSORT: Describe the interventions for each group with sufficient details to allow replication, including how and when they were actually administered**		
**5-i) Mention names, credential, affiliations of the developers, sponsors, and owners**		
A previously published methods paper in CCT reports provides more details. The algorithm that drives the app was developed by the investigator (LE Burke) and the app was developed by members of the investigative team at the University of Pittsburgh.		
**5-ii) Describe the history/development process**		
"We previously examined the effect of providing feedback to dietary SM and PA; however, the hardware and software used was rudimentary compared to today's technology.[17] Despite those limitations, remotely delivered FB messages enhanced SM adherence and improved weight loss.[31, 38] Those results and significant mobile technology enhancements provided groundwork for the expanded algorithm and FB intervention used in the current trial, SMARTER.[25]"<br>The basic algorithm was developed several years ago and used in a RCT during the period between 2005 and 2010 (SMART trial published in several papers, Burke et al.) At that time, we had to use someone in industry to develop the feedback system based on a much smaller algorithm.		
**5-iii) Revisions and updating**		
As addressed above, a simpler version of the algorithm was developed several years ago and we used industry to send the messages to participants on personal digital assistant s(Palm Pilot). We did not have an "app" at that time.		
**5-iv) Quality assurance methods**		
The rigor of the study design and data collection methods as well as the analytic methods are addressed throughout the design and methods paper published in CCT (Burke et al., 2021, Contemporary Clinical Trials 91. doi.org/10.1016/j.cct.2020.105958) and in the current manuscript.		
**5-v) Ensure replicability by publishing the source code, and/or providing screenshots/screen-capture video, and/or providing flowcharts of the algorithms used**		
Samples of the algorithm have been published previously, as noted in CCT.		
**5-vi) Digital preservation**		
The above information is archived at the University of Pittsburgh. A second methods paper describing the details of the digital component of the study is in progress.		
**5-vii) Access**		
Upon randomization, "only the participants in the SM+FB group were given the code to access the app in the app store." Once the study was completed, the app is no longer available in the app store.		
**5-viii) Mode of delivery, features/functionalities/components of the intervention and comparator, and the theoretical framework**		
"Feedback messages. The feedback algorithm was programmed on the study's server and used real-time synced SM data to send the FB message up to three times per day. Individuals in SM+FB group received up to three FB messages per day on their smartphone during waking hours tailored to the most recent SM data and addressing caloric, fat and added-sugar intake daily and PA every other day. Weekly weight FB was based on whether self-weighing occurred and the amount/rate of weight change. FB messages addressed one behavior at a time. If the message was not opened within one hour of being sent, it disappeared; if the message was opened, the participant could save it for future review."<br><br>"Engagement with SM tools was a crucial component of the intervention as the algorithm used the SM data to determine an appropriate FB message. If the participant did not SM, FB messages encouraged SM. After 2 weeks of missing SM data, staff sent an email query about technical issues and encouraged SM. Additional details on the algorithm and FB messages are published elsewhere.25 The message library was changed at least monthly to avoid participant desensitization to FB.31 Individuals in the SM group did not receive FB messages or staff emails. Further details of all intervention components and the algorithm driving the feedback messages have been published elsewhere."		
**5-ix) Describe use parameters**		
As above, the feedback messages were programmed to be sent within 3 separate periods of the day: morning, afternoon and evening.		
**5-x) Clarify the level of human involvement**		
There was no human involvement in the delivery of the feedback messages. It was automatically driven by the algorithm.		
**5-xi) Report any prompts/reminders used**		
"The participant received a prompt that there was a new FB message on the smartphone, if it was not opened within one hour of being sent, it disappeared; if the message was opened, the participant could save it for future review.<br>If the participant did not SM, FB messages encouraged SM. After 2 weeks of missing SM data, staff sent an email query about technical issues and encouraged SM. "		
**5-xii) Describe any co-interventions (incl. training/support)**		
There were no co-interventions. Everyone in the trial had a smartphone (their own), and study provided Fitbit and smart scale that they were instructed to use daily. The only difference between the two groups was the provision of feedback message.		
**6a) CONSORT: Completely defined pre-specified primary and secondary outcome measures, including how and when they were assessed**		
Outcomes measures are addressed in the paper: primary outcome, percent weight change from baseline to 12 months. Secondary measures are percent of days adherent to the dietary self-monitoring and dietary goal and percent of feedback messages sent that were opened.		
**6a-i) Online questionnaires: describe if they were validated for online use and apply CHERRIES items to describe how the questionnaires were designed/deployed**		
All online surveys used in this trial are standardized measures that have been used by our investigative for several studies and thus validated in our population.		
**6a-ii) Describe whether and how "use" (including intensity of use/dosage) was defined/measured/monitored**		
The intervention dose was preset at 3 feedback messages per day. If there was a variation in the number of messages delivered, it was dur to the lack of engagement by the participant in the use of the devices and not self-monitoring, or not opening the messages.		
**6a-iii) Describe whether, how, and when qualitative feedback from participants was obtained**		
A focus group study was conducted with participants after study completion. These data are in the final stage of analysis.		
**6b) CONSORT: Any changes to trial outcomes after the trial commenced, with reasons**		
"We used a combination of online and in-person recruitment/screening procedures. Initial screening of eligibility criteria was conducted nline with online consent form to collect these data. For the verification of weight/height for the calculation of body mass index (BMI) we conducted in-person assessment.:		
**7a) CONSORT: How sample size was determined**		
**7a-i) Describe whether and how expected attrition was taken into account when calculating the sample size**		
Sample size. The planned total sample size for this RCT was determined as 530 (265 per treatment group) allowing for 0.80 statistical power to detect effect sizes (standardized mean differences, d) as small as d=0.301 for the mean percent weight changes at 6 and 12 months between the SM computed and SM + FB groups when using linear mixed modeling with linear contrasts at a Bonferroni-adjusted significance level of .025 and for at most 20% attrition. [25]. Due to the COVID-19 pandemic, recruitment was stopped in March 2020 with 502 randomized (251 per treatment arm). With this reduced sample size, slightly larger small to medium effect sizes of d=0.309 would still be detectable with 0.80 power at an adjusted significance level of .025, allowing for up to 20% attrition.		
**7b) CONSORT: When applicable, explanation of any interim analyses and stopping guidelines**		
Outcomes measures are addressed in the paper: primary outcome, percent weight change from baseline to 12 months. Secondary measures are percent of days adherent to the dietary self-monitoring and dietary goal and percent of feedback messages sent that were opened.		
**8a) CONSORT: Method used to generate the random allocation sequence**		
" research staff used a randomization software program to determine group assignment that was generated using minimization with stratification by gender (male/female) and race (Black/non-Black) with equal allocation to the two treatment conditions. See Figure 1, CONSORT Diagram."		
**8b) CONSORT: Type of randomisation; details of any restriction (such as blocking and block size)**		

"After completing the intervention consent, research staff used a randomization software program to determine group assignment that was generated using minimization with stratification by gender (male/female) and race (Black/non-Black) with equal allocation to the two treatment conditions. See Figure 1, CONSORT Diagram."		
**9) CONSORT: Mechanism used to implement the random allocation sequence (such as sequentially numbered containers), describing any steps taken to conceal the sequence until interventions were assigned**		
"...staff used a randomization software program to determine group assignment..."		
**10) CONSORT: Who generated the random allocation sequence, who enrolled participants, and who assigned participants to interventions**		
The trial statistician developed the random allocation sequence using a randomization software. "The trained research ran the program to determine the treatment assignment. using minimization with stratification by gender (male/female) and race (Black/non-Black) with equal allocation to the two treatment conditions."		
**11a) CONSORT: Blinding - If done, who was blinded after assignment to interventions (for example, participants, care providers, those assessing outcomes) and how**		
**11a-i) Specify who was blinded, and who wasn't**		
"Key staff who conducted the assessments were not blinded to the treatment assignment, whereas all other personnel and investigators, including the statisticians, were blinded to assignment. Since participants were informed of both treatment conditions they could not be blinded. "		
**11a-ii) Discuss e.g., whether participants knew which intervention was the "intervention of interest" and which one was the "comparator"**		
It is not addressed in the paper but via the informed consent process, participants knew the difference between the two interventions.		
**11b) CONSORT: If relevant, description of the similarity of interventions**		
It is not addressed in the paper but via the informed consent process, participants knew the difference between the two interventions.		
**12a) CONSORT: Statistical methods used to compare groups for primary and secondary outcomes**		
"Continuous variables were summarized as mean ± SD, and descriptive statistics for categorical variables were reported as counts (%). Appropriate group comparative analyses were performed on participant descriptors and outcome variables at baseline by randomized treatment assignment.[39] The effect of treatment assignment on percent weight change over 12 months was examined using linear mixed modeling following intention-to-treat. Models included random intercept and unstructured variance-covariance matrix for the repeated assessments, supported by Akaike's Information and Bayesian Information Criteria. The base model included fixed effects for time (baseline vs. 6 months and 12 months), group (SM+FB vs. SM alone), time (baseline vs. 6 months and 12 months) and group by time interaction.<br>The effect of the percentage of FB messages opened on percent weight change from baseline to 12 months for the SM+FB group was analyzed using univariate linear regression. Additionally, the associations of monthly percentage days adherent to the calorie goal with treatment assignment and the percentage of FB messages opened were analyzed using separate linear mixed models with random intercept and slope for the total sample and for the SM+FB group, respectively. We conducted sensitivity analyses on the treatment effects on monthly percentages of days adherent to the calorie goal over 12 months in the total sample and on the associations between monthly percentages of FB messages opened and monthly percentages of days adherent to the calorie goal in the SM+FB group for the varying monthly percentage of days with sufficient dietary SM data (data not shown). Here we report the results using days with ≥50% of the calorie goal recorded, or ≥15 of 30 days with sufficient dietary SM data.<br>Model assessment (i.e., residual analyses with influence diagnostics) was performed for each fitted model; sensitivity analyses were conducted for outlying/influential observations, and to explore the effect of the COVID-19 pandemic on the efficacy of treatment assignment on percent weight change (data not shown). All analyses were performed using SAS version 9.4 [SAS Institute Inc., Cary, NC, USA]."		
**12a-i) Imputation techniques to deal with attrition / missing values**		
Missing weight values were imputed using self-monitoring data from the Wi-Fi scale being used for weight self-monitoring at home. If the weight self-monitoring data were more than ±14 days from the projected 6-month date, 0.30 kg/month or 0.01 kg/day weight gain was assumed from the last available self-weighing value (Wadden et al, Arch Int Med 2001)		
**12b) CONSORT: Methods for additional analyses, such as subgroup analyses and adjusted analyses**		
All analyses conducted described above.		
RESULTS		
**13a) CONSORT: For each group, the numbers of participants who were randomly assigned, received intended treatment, and were analysed for the primary outcome**		
All of this information is in the detail CONSORT figure, Figure 1 in the paper.		
**13b) CONSORT: For each group, losses and exclusions after randomisation, together with reasons**		
The randomized sample was 502. At 12 months, overall retention was 78.5%. Retention was similar by treatment condition, SM+FB (80.5%) and SM (76.5%) (X2 =1.18, P=.277).		
**13b-i) Attrition diagram**		
At 12 months, overall retention was 78.5%. Retention was similar by treatment condition, SM+FB (80.5%) and SM (76.5%) (X2 =1.18, P=.277). See CONSORT figure for full details.		
**14a) CONSORT: Dates defining the periods of recruitment and follow-up**		
Recruitment, conducted in the greater community surrounding Pittsburgh, PA, commenced in August 2018 and ended in March 2020. The intervention trial was completed in April 2021		
**14a-i) Indicate if critical "secular events" fell into the study period**		
On March 16, 2020, the university shutdown all in-person encounters with study participants. Therefore, we abruptly ended recruitment, screening and enrollment procedures after March 16, 2020 (i.e., COVID-19 pandemic shutdown start). We collected 12-month weight data remotely from the participant's study-provided scale, which assessed only weight and percent fat mass. Staff contacted participants to instruct them to dress in clothing like the baseline assessment and report their weight, which was also captured electronically.[25]		
**14b) CONSORT: Why the trial ended or was stopped (early)**		
No, the trial was not ended early.		
**15) CONSORT: A table showing baseline demographic and clinical characteristics for each group**		
The CONSORT figure shows the number of eligible and ineligible individuals and the reason for exclusion.		
**15-i) Report demographics associated with digital divide issues**		
We did not find differences in primary outcomes by these groups at this point. We are in the early phase of examining in detail the engagement of participants in all components of the study protocol, which may shed light on the digital divide effect if there was any in our study.		
**16a) CONSORT: For each group, number of participants (denominator) included in each analysis and whether the analysis was by original assigned groups**		
**16-i) Report multiple "denominators" and provide definitions**		
At baseline, 500 participants had complete body fat data. At 6 months, 338 participants had body fat data, and at 12 months, 186 participants had data.<br>At baseline, all male participants (n=103) had complete waist circumference data. At 6 months, 69 males had waist circumference data, and at 12 months, 37 males had data.<br>At baseline, all participants (N=502) had complete blood pressure data. At 6 months, 340 participants had blood pressure data, and at 12 months, 189 participants had data.<br>At baseline, all female participants (n=399) had complete waist circumference data. At 6 months, 271 females had waist circumference data, and at 12 months, 149 females had data.		
**16-ii) Primary analysis should be intent-to-treat**		
"The effect of treatment assignment on percent weight change over 12 months was examined using linear mixed modeling following intention-to-treat."		
**17a) CONSORT: For each primary and secondary outcome, results for each group, and the estimated effect size and its precision (such as 95% confidence interval)**		
"Confidence intervals were provided for all statistical results. For example..."On average both groups had statistically significant weight loss over 12 months (b6_months=-2.94; 95% CI -3.70 to -2.19; b12_months=-2.34; 95% CI -3.10 to -1.59; F= 61.46, P<.0001). "		
**17a-i) Presentation of process outcomes such as metrics of use and intensity of use**		
The process variable of adherence to self-monitoring was examined briefly as this is the focus of another major paper. "The effect of the percentage of FB messages opened on percent weight change from baseline to 12 months for the SM+FB group was analyzed using univariate linear regression. Additionally, the associations of monthly percentage days adherent to the calorie goal with treatment assignment and the percentage of FB messages opened were analyzed using separate linear mixed models with random intercept and slope for the total sample and for the SM+FB group, respectively. We conducted sensitivity analyses on the treatment effects on monthly percentages of days adherent to the calorie goal over 12 months in the total sample and on the associations between monthly percentages of FB messages opened and monthly percentages of days adherent to the calorie goal in the SM+FB group for the varying monthly percentage of days with sufficient dietary SM data (data not shown). Here we report the results using days with ≥50% of the calorie goal recorded, or ≥15 of 30 days with sufficient dietary SM data."		
**17b) CONSORT: For binary outcomes, presentation of both absolute and relative effect sizes is recommended**		
We do not report binary outcomes.		
**18) CONSORT: Results of any other analyses performed, including subgroup analyses and adjusted analyses, distinguishing pre-specified from exploratory**		
Analyses were conducted in the intervention group of the effect of opening the feedback messages.... "In the SM+FB arm, median [inter-quartile range] percentage of FB messages received from baseline to 12 months was 42.19% [45.30] and ranged from 1.28% to 93.70%."		
**18-i) Subgroup analysis of comparing only users**		
Analyses were not conducted examining only users.		

**19) CONSORT: All important harms or unintended effects in each group**		
No unintended effects were observed or reported.		
**19-i) Include privacy breaches, technical problems**		
None were observed or reported.		
**19-ii) Include qualitative feedback from participants or observations from staff/researchers**		
This paper focuses on the primary outcome of the RCT: percent weight change and effects of intervention (opening feedback messages) on the outcome. These other topics (qualitative interviews) are not the focus of this paper.		
DISCUSSION		
**20) CONSORT: Trial limitations, addressing sources of potential bias, imprecision, multiplicity of analyses**		
**20-i) Typical limitations in ehealth trials**		
"Limitations include recruitment of fewer males and minorities than targeted, which limits generalizability. The COVID-19 shutdown ended all in-person interactions including assessments which may have affected engagement. "		
**21) CONSORT: Generalisability (external validity, applicability) of the trial findings**		
**21-i) Generalizability to other populations**		
"...recruitment of fewer males and minorities than targeted, which limits generalizability"		
**21-ii) Discuss if there were elements in the RCT that would be different in a routine application setting**		
"It is possible that the inclusion of standard behavioral treatment with in-person contact and group sessions or individual coaching would have had a significant impact on adherence to SM and engagement with the devices. Studies that have included in-person coaching have reported larger weight losses. However, these studies do not address the key issues addressing the high rates of obesity prevalence and the limited access to weight loss programs, and also the issue we addressed of reducing burden and cost while increasing scalability."		
**22) CONSORT: Interpretation consistent with results, balancing benefits and harms, and considering other relevant evidence**		
**22-i) Restate study questions and summarize the answers suggested by the data, starting with primary outcomes and process outcomes (use)**		
"We conducted a trial of a scalable, remotely delivered behavioral weight loss intervention and tested the efficacy of a custom-developed, theoretically-based smartphone app that provided real-time FB remotely to reinforce diet, PA, and self-weighing behaviors. We observed a small but significant percent weight change from baseline to 12 months with no significant difference between the groups"		
**22-ii) Highlight unanswered new questions, suggest future research**		
"...it is difficult to ascertain how much of the human interventionist component can be replaced to make weight loss treatments scalable to a broader reach and lower operational costs. This critical gap in the evidence needs to be addressed in future studies so we can broaden our reach to the millions who need weight loss treatment, particularly those who do not have access to existing clinical and commercial weight loss programs. "		
Other information		
**23) CONSORT: Registration number and name of trial registry**		
The study is registered with Clinicaltrials.gov (NCT03367936).		
**24) CONSORT: Where the full trial protocol can be accessed, if available**		
The full IRB approved protocol was uploaded to the Multimedia Materials section. . A detailed methods paper was published in Burke et al., CCT, 2020.		
**25) CONSORT: Sources of funding and other support (such as supply of drugs), role of funders**		
25 NIH R01-HL131583 (LE Burke), R01-HL131583S (J Kariuki), and F31-HL156278 (J Cheng) and the University of Pittsburgh Clinical Translational Science Institute (UL1-TR001857 (S Dr. Steven Reis).		
**X26-i) Comment on ethics committee approval**		
The study was approved by the Institutional Review Board at the University of Pittsburgh and registered on ClinicalTrials.gov (NCT03367936). Research staff informed all participants of screening procedures prior to obtaining consent and performed in-person informed consent for the intervention study."		
**x26-ii) Outline informed consent procedures**		
Trained staff used the informed consent to walk the participant through the study intervention and comparator group.		
**X26-iii) Safety and security procedures**		
Details of security and safety measures are in the design paper.		
**X27-i) State the relation of the study team towards the system being evaluated**		
No authors/investigative team members have any conflicts to disclose.		
